# Assessment of autonomic function by long-term heart rate variability: beyond the classical framework of LF and HF measurements

**DOI:** 10.1186/s40101-021-00272-y

**Published:** 2021-11-30

**Authors:** Junichiro Hayano, Emi Yuda

**Affiliations:** 1 Heart Beat Science Lab, Co., Ltd., Aoba 6-6-40 Aramaki Aoba-ku, Sendai, 980-0845 Japan; 2 Nagoya City University, Kawasumi 1, Mizuho-cho Mizuho-ku, Nagoya, 467-8602 Japan; 3 Center for Data-Driven Science and Artificial Intelligence, Tohoku University, 41 Kawauchi, Aoba-ku, Sendai, 980-8576 Japan

**Keywords:** Autonomic nervous system, Baroreceptor reflex, Cyclic variation of heart rate, Heart rate turbulence, Heart rate variability, Mortality, Pulse rate variability, Pulse wave, Photoplethysmography, Risk stratification, Sleep apnea, Sympathetic nervous system

## Abstract

In the assessment of autonomic function by heart rate variability (HRV), the framework that the power of high-frequency component or its surrogate indices reflects parasympathetic activity, while the power of low-frequency component or LF/HF reflects sympathetic activity has been used as the theoretical basis for the interpretation of HRV. Although this *classical* framework has contributed greatly to the widespread use of HRV for the assessment of autonomic function, it was obtained from studies of short-term HRV (typically 5‑10 min) under tightly controlled conditions. If it is applied to long-term HRV (typically 24 h) under free-running conditions in daily life, erroneous conclusions could be drawn. Also, long-term HRV could contain untapped useful information that is not revealed in the classical framework. In this review, we discuss the limitations of the classical framework and present studies that extracted autonomic function indicators and other useful biomedical information from long-term HRV using novel approaches beyond the classical framework. Those methods include non-Gaussianity index, HRV sleep index, heart rate turbulence, and the frequency and amplitude of cyclic variation of heart rate.

## Introduction

Although the analysis of HRV is widely used in various fields as a non-invasive assessment of autonomic function, it has the potential to draw inappropriate conclusions when applied beyond its limitations. This is mainly due to the uncritical use of HRV interpretations based on the simple framework that the power of high-frequency (HF, 0.15‑0.4 Hz) HRV component or its surrogate indices (rMSSD and pNN50) reflects parasympathetic activity, while the relative power of low-frequency (LF, 0.04‑0.15 Hz) component or LF-to-HF power ratio (LF/HF) reflects sympathetic activity. This *classical* framework has been derived from studies of short-term HRV (typically 5‑10 min) under tightly controlled conditions of measurement environment, body position, physical activity, and respiratory state [1–3]. Consequently, applying the framework to HRV where these conditions are not met, especially long-term HRV (typically 24 h) obtained under free-running conditions with wearable sensors, may lead to erroneous conclusions, and also prevent the proper extraction of useful information contained in the HRV. The use of the classical framework needs to be more strictly limited, and it is an important issue for the development of HRV researches [4].

Long-term HRV under free-running conditions is thought to consist of at least five components of variability: first, circadian and ultradian rhythms, including LF and HF components; second, 1/*f* or fractal fluctuation generated by complex neural networks in the brain [5, 6]; third, variability caused by various daily physical and mental activities as well as weather and indoor environmental parameters; fourth, variability caused by the cardiac pacemaker system itself, including heart rate fragmentation (HRF) [7–9]; and fifth, variability in sinus rhythm caused by spontaneous accidental events such as extrasystoles and sleep apnea. In this review, we will first describe the problems that could arise in applying the classical framework to such long-term HRV and then studies that have extracted autonomic function indicators and other useful biomedical information using novel approaches beyond the classical framework.

### Limitations of classical framework

The evidence supporting the classical framework of HRV is as follows. The facts for the relationship between HF power and cardiac vagal activity include that (1) transfer function analysis of autonomic heart rate controls in an isolated canine heart model shows that the LF component of HRV is mediated by both sympathetic and vagus nerves, while the HF component is mediated solely by the vagus nerves [10], (2) the HF component disappears when the cardiac vagus nerves are blocked either physically by heart transplantation [11] or pharmacologically by high-dose atropine [1, 12], and (3) in healthy young subjects, there is a proportional relationship between the HF amplitude under paced breathing and the cardiac vagal control that is measured as the change in mean R-R interval with pharmacological vagal blockade under complete β-adrenergic blockade [3]. The facts for the relationship between LF component and sympathetic activity include that (1) the increase in LF power with standing or head-up tilting, if any, is abolished by beta-blockers [1, 13] and (2) the relative LF power and LF/HF increases consistently with standing and head-up tilt [2].

The uncritical application of this classical framework to long-term HRV under free-running conditions may lead to erroneous conclusions. A clear example is the relationship between long-term HRV and prognosis after acute myocardial infarction (MI). Decreased long-term HRV is a predictor of increased risk of post-MI mortality, and this association is explained by the detrimental effects of cardiac vagal dysfunction. However, among the spectral components of long-term HRV, including ultra-low-frequency (ULF, < 0.00033 Hz), very low frequency (VLF, 0.0033‑0.04 Hz), and LF components, the predictive power of decreased HF is the lowest [14]. In addition, even though increased sympathetic activity is a well-known risk of mortality in post-MI patients, in long-term HRV, the lower the LF/HF, the higher the risk of mortality [15, 16]. These are mainly due to the fact that patients with a better prognosis in general have a higher level of physical activity and spend more time in a standing position in daily life, which may decrease HF power and increase LF/HF on average. If these observations are interpreted in a classical framework, they negate the adverse effects of autonomic dysfunction in post-MI pathophysiology.

Even when applied to short-term HRV, the classical framework has several important limitations. The link between the HF component and cardiac parasympathetic activity is due to the association between the magnitude of respiratory sinus arrhythmia and cardiac parasympathetic activity [17, 18], which requires that the respiratory rate is always maintained in the range of 9‑24 beats/min (0.15‑0.4 Hz). Even within this range, the magnitude of respiratory sinus arrhythmia is inversely proportional to the respiratory rate, independent of cardiac parasympathetic activity [19, 20]. In addition to respiratory sinus arrhythmias, the contaminations of transient atrial fibrillation or other non-autonomically mediated HRV, such as HRF [7–9], may also increase the apparent HF power.

There are important limitations to the assessment of autonomic function by HRV that should be better recognized. It should be noted, however, that these limitations are for the evaluation of autonomic function by HRV based on the classical framework, not for the HRV analysis itself.

### Assessment of sympathetic function by HRV

Although many studies have used the relative LF power or LF/HF as an index of sympathetic activity or sympathetic predominance in sympatho-vagal balance, the only evidence that supports this interpretation is the postural increase of these indices, and on the contrary, much evidence has been accumulated to reject this hypothesis. Studies using sympathetic indices such as muscle sympathetic activity [21, 22] and positron emission tomographic neuroimaging [23, 24] have rejected any relationship between LF power or LF/HF and sympathetic activity. As long as the classical framework is used, reliable assessment of sympathetic function by HRV should be considered impossible. So, does HRV not contain information about sympathetic nervous activity? Probably it does. Two examples that suggest this are the relationship between individual differences in the postural LF response of short-term HRV and prognosis, and the relationship between the non-Gaussian index *λ* of long-term HRV and prognosis of cardiovascular diseases.

#### LF rise of short-term HRV

**Fig. 1:**
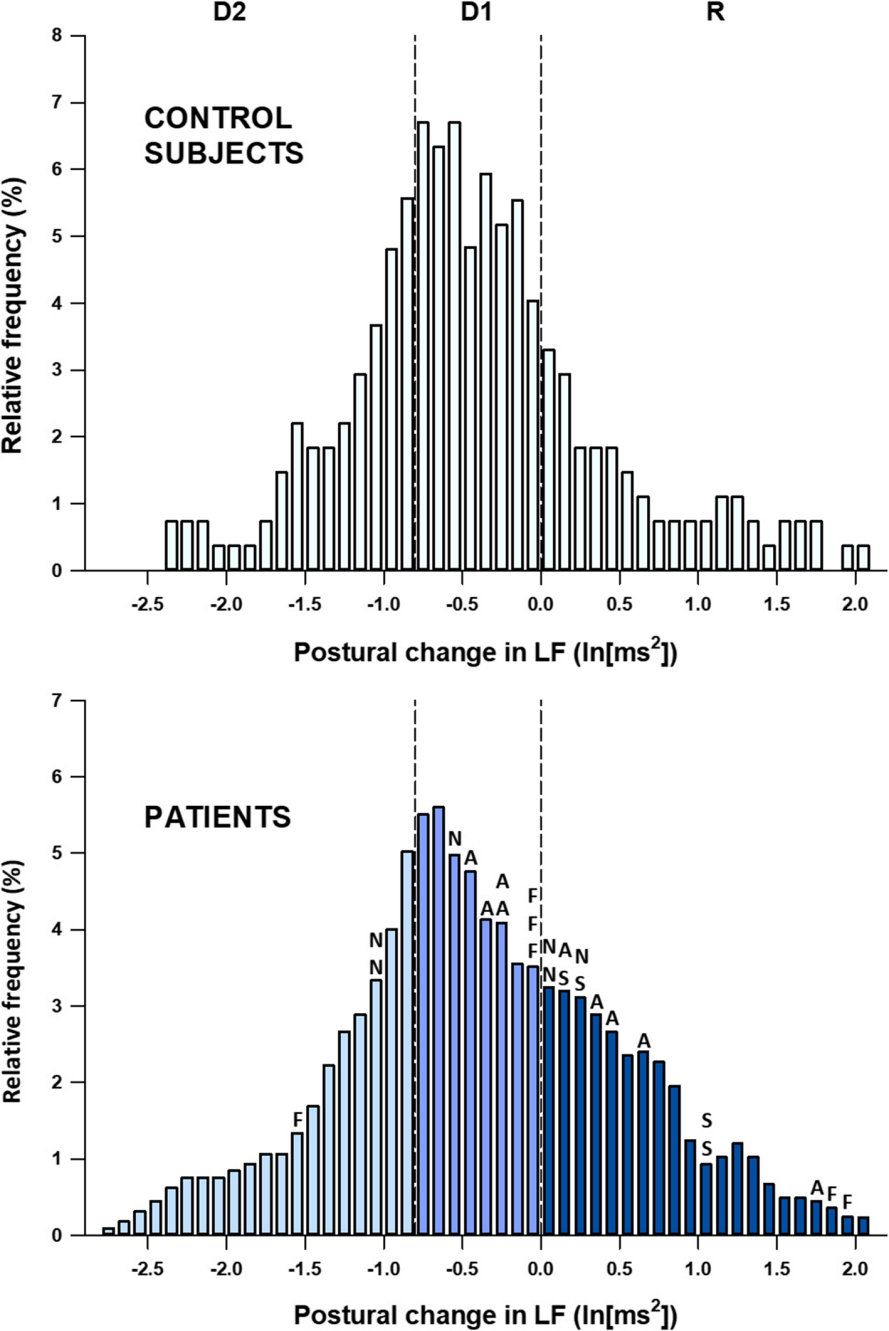
Distributions in changes in LF power with head-up tilting among 90 healthy subjects (upper panel) and among 250 patients undergoing coronary angiography (lower panel). Vertical-dashed lines indicate the cutoff points for the trisection of the LF response into large drop (D2, *n* = 82), small drop (D1, *n* = 83), and rise (R, *n* = 85) in angiographic patients. With the same cutoff, healthy subjects divided into D2 (*n* = 28), D1 (41), and R (*n* = 21). Out of 250 patients, 25 died during 99 months of follow-up. Letters at the top of the bars in the upper panel indicate the cause of death of individual patients: A, acute myocardial infarction (MI); F, fatal stroke; N, noncardiac causes; S, sudden cardiac death. Modified Fig. 2 of reference [25]

Previous studies have reported that standing or head-up tilt increases LF power in normal subjects under spontaneous breathing [1], but LF power under paced breathing increases in a part of normal subjects (about 20%) and remains unchanged or even decreases in the rest of subjects (Fig. 1 upper panel) [25].

In a prospective study of the prognostic value of short-term HRV in patients with stable coronary artery disease (CAD) [25], we analyzed postural LF response to head-up tilting under paced breathing in 250 patients who underwent elective coronary angiography. A postural increase in LF power (LF rise) was observed in 85 (34%) patients (group R), while a small drop was observed in 83 (33%) patients (group D1) and a large drop in 82 (33%) patients (group D2). During a subsequent follow-up period of 99 months, there were 13 cardiac deaths and 12 noncardiac deaths (Fig. 1 lower panel). The three groups did not differ in terms of clinical features or CAD severity at baseline or coronary interventions during the follow-up period; however, cardiac mortality rates during the 99 months were 12%, 6%, and 0% in groups R, D1, and D2, respectively. The difference was enhanced when analyzed excluding 64 patients treated with β-blockers during the follow-up period (15%, 7%, and 0%, respectively). These observations indicate that the LF rise is a predictor of cardiac mortality risk in patients with stable CAD.

Together with previous observations that β-adrenergic blockade suppresses LF rise [1, 13], LF rise is a marker of posture-induced sympathetic overactivation, which may lead to poor prognosis in patients with stable CAD. However, the LF component in upright posture is mediated by both sympathetic and parasympathetic nerves [1]. Thus, LF rise could be due partly to a decrease in posture-induced decline in parasympathetic activity, which in turn could be due to a decrease in parasympathetic response reserve in the supine position. For either case, LF rise reflects greater dependence on sympathetic activation than on vagal withdrawal in the autonomic neural regulation of the postural heart rate response.

#### Non-Gaussianity of long-term HRV

**Fig. 2:**
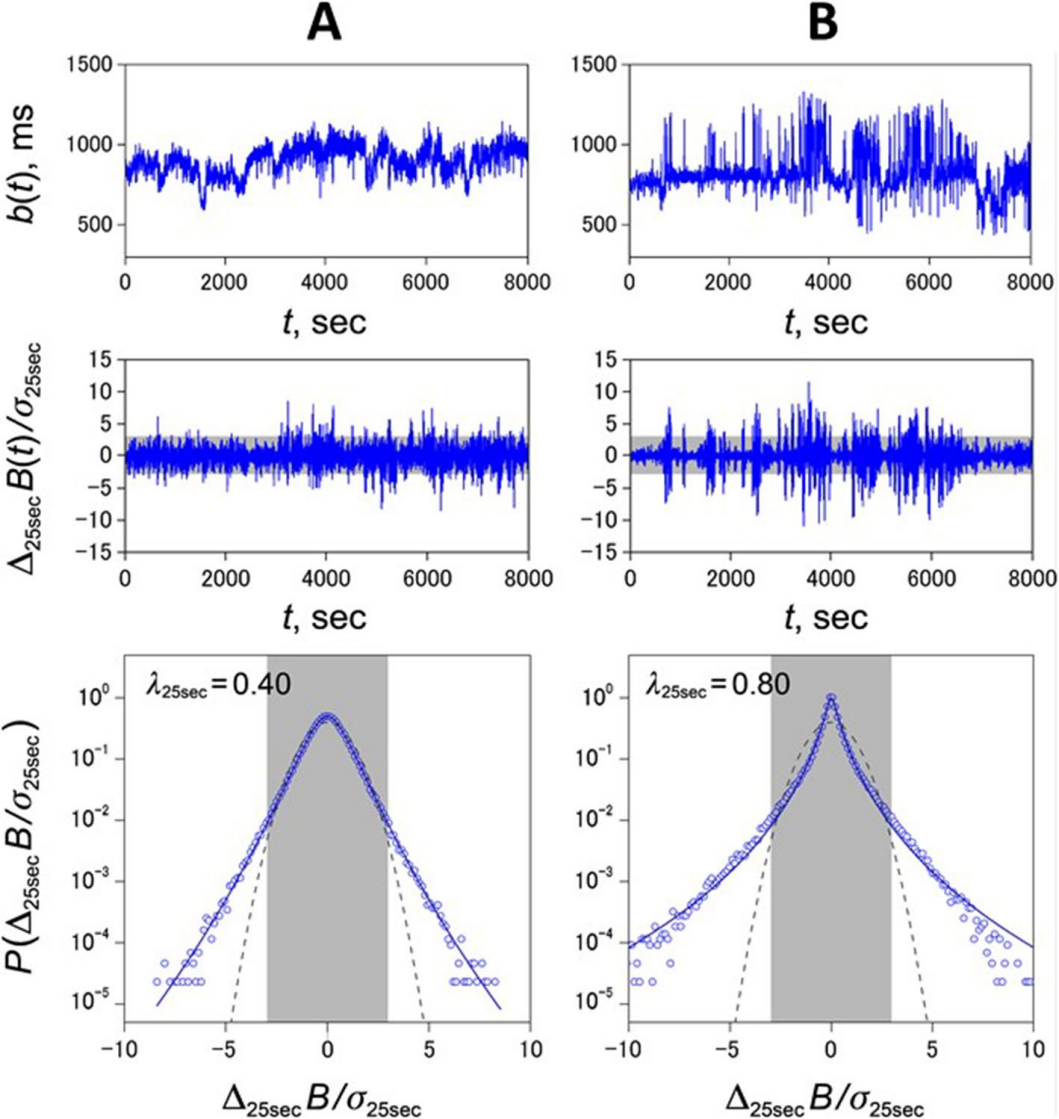
Analysis of non-Gaussian heart rate fluctuations in two representative post-MI patients: survivor (**A**) and cardiac death (**B**). From the top, trend graphs of normal-to-normal R-R interval *b*(*t*), standardized time series of heart rate increments Δ_25s_*B*(*t*), and standardized probability density functions (PDFs) of heart rate increments *P*(Δ_25s_*B*(*t*)) with non-Gaussianity index of λ_25s_. In the middle- and bottom-row panels, gray-shaded areas cover ± 3 SD ranges. In the bottom-row panels, solid lines indicate the PDF approximated for the corresponding λ_25s_ values by a non-Gaussian model [27]. Compared to the survivor, bursty changes with amplitudes exceeding ±3 SD (gray-shaded areas in the middle-row panels) increase in the cardiac death patient, resulting in a sharper peak with fatter tails in the PDF (bottom-row panels) and a larger value of λ_25s_, which reflects the degree of deviation from the Gaussian distribution (dashed lines in the bottom-row panels). Modified Fig. 1 of reference [30]

Non-Gaussianity index (*λ*) is an index of long-term HRV developed by Kiyono et al. [26–28]. It characterizes increased probability of the large abrupt heart rate deviations from its trend (Fig. 2). To calculate the *λ*, in the detrended instantaneous heart rate time series, the increments of heart rate (the difference between two heart rates apart 25 s [in case of *λ*_25s_]; when the heart rate decreases, the increment is negative) are measured at all time points. Then, the relationship between the magnitude of the heart rate increment and its probability of occurrence is expressed as a probability density function (PDF). The PDF of the heart rate increment is known to show a non-Gaussian distribution, and *λ* represents the degree of deviation from the Gaussian distribution. The *λ* is larger for PDFs with a more peaked center and fatter tails. The fatter tails of the PDF indicate that the more frequent large abrupt changes in heart rate occur, compared to smaller changes.

Studies of long-term HRV have reported an association between increased non-Gaussianity of HRV and increased risk of mortality in patients with congestive heart failure (CHF) [29] and in patients after MI [30] particularly those with preserved left ventricular ejection fraction [31]. In these and other studies, *λ* showed unique properties that differentiate it from other HRV indices [32]. First, for the major time-domain HRV indices (SDNN, rMSSD, HRV triangular index [33], deceleration capacity [34], etc.) and frequency-domain HRV indices (ULF, VLF, LF, LF/HF, etc.), their decreases predict mortality risk, whereas for *λ*, its increase predicts mortality risk [29, 30]. Second, in a big data analysis (*n* = 265,291) for the redundancy among long-term HRV indices, traditional long-term HRV indices (SDNN, VLF, deceleration capacity, and scaling exponent *α*_1_ [6, 16]) showed high similarity (assessed by mutual explained variance) and formed a single cluster, whereas *λ*_25s_ had the lowest similarity with other HRV indices and was located far from the clusters of other indices [32]. These traditional HRV indices primarily reflect vagal function [33–35]. Third, although the predictive power of traditional HRV indices for mortality risk in patients with CHF is low to moderate at best [36–39], *λ* detects fundamental characteristics of HRV in CHF and its increase significantly and independently reflect the increased risk for death in these patients [29]. Fourth, although decreases in conventional long-term HRV indices [16, 34, 40] and other indices of parasympathetic dysfunctions [15, 41] predict increased risk of both cardiac and non-cardiac deaths in post-MI patients, increased *λ*_25s_ predicts exclusively cardiac death but not non-cardiac death [30]. Finally, *λ*_25s_ is lower in post-MI patients taking β-blockers compared with those not taking β-blockers [30]. In CHF, a state of sympathetic cardiac overdrive, there is an increase in *λ*_25s_ along with a decrease in the HRV index reflecting vagal dysfunction, whereas in multiple system atrophy, a neurodegenerative disorder associated with preganglionic sympathetic failure [42] and Parkinson’s disease, which is often accompanied by postganglionic sympathetic failure [43, 44], there is a decrease in the HRV index reflecting vagal dysfunction, but no increase in λ_25s_ [28]. These facts indicate that an increase in non-Gaussianity index *λ*_25s_ of long-term HRV may be a marker of sympathetic cardiac overdrive.

### Analysis of HRV associated with physiological events

During long-term monitoring, both physiological and pathophysiological events may occur incidentally and leave a footprint on HRV. HRV indices derived from the classical framework may also be affected by such events, but the changes are often non-specific and not useful for their accurate detection. Several methods of HRV analysis are known to detect such specific events.

#### Estimation of sleep stage by HRV

The quality and quantity of sleep is an important factor in healthcare, but its evaluation often relies on subjective self-assessment. It is desirable to have objective indices to estimate accurate durations of sleep and preferably of sleep stages in daily life. In this regard, several researchers have attempted to detect sleep and determine sleep stages by analyzing long-term HRV [45–50]. Since it is generally believed that the transition from wakefulness to sleep, especially to non-REM (NREM) sleep, is accompanied by sympathetic inhibition and parasympathetic activation, models using autonomic indices of HRV based on the classical framework have been proposed. However, the univariate predictive power of these indices is not sufficient and there is no physiological basis to explain why they distinguish light sleep from awake rest.

**Fig. 3:**
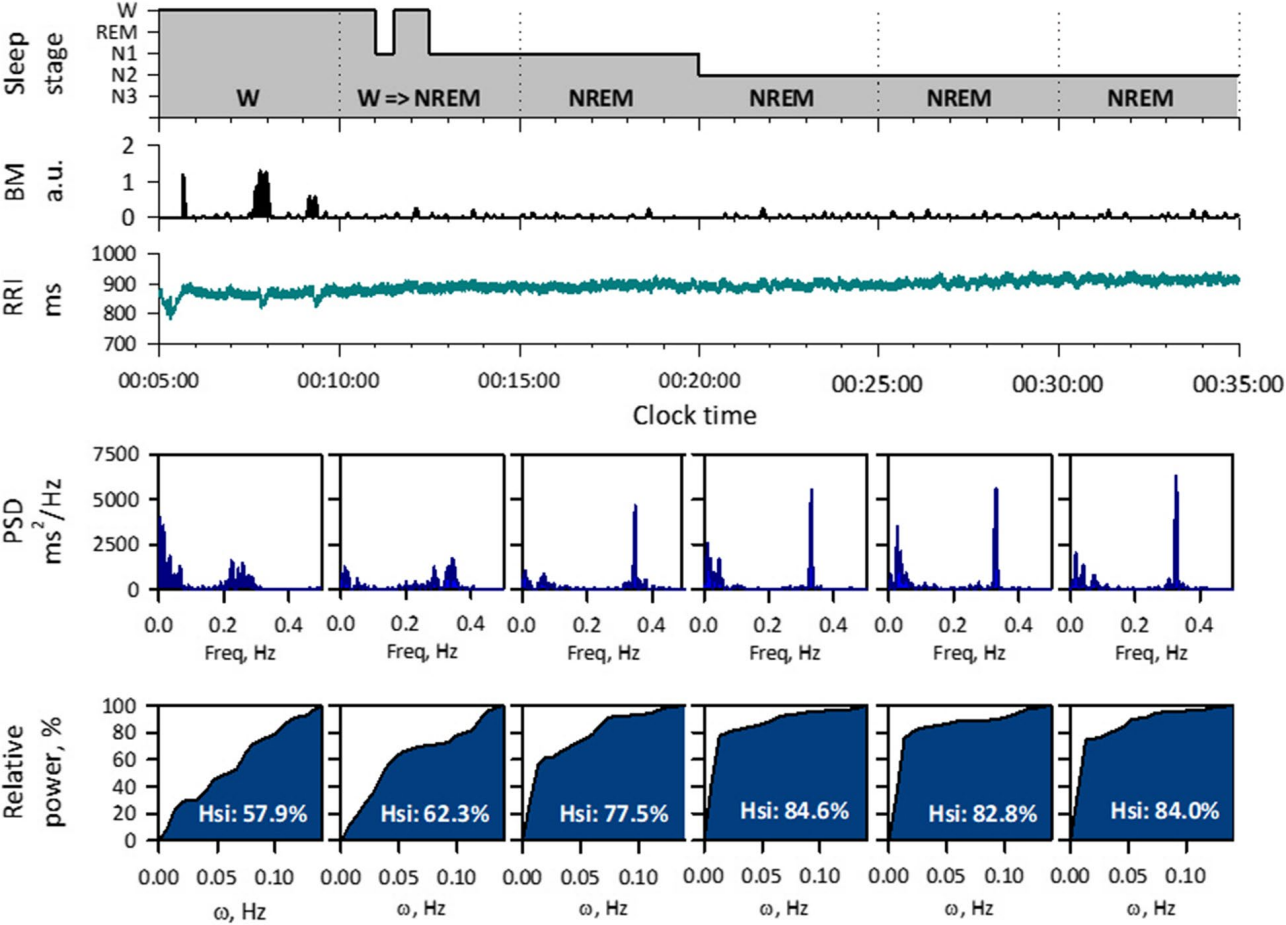
Detection of sleep onset by HRV during polysomnography in a healthy male subject. From top: hypnogram, body motion (BM) by actigraphy, R-R interval (RRI), power spectral density (PSD), and relative power of HF to the integrated width (ω) of the frequency band around the HF peak. When the sleep stage changes from wakefulness (W) to NREM (N1, N2, N3), the large BM disappears, a sharp peak appears in the HF band (0.15‑0.4 Hz) of the RRI power spectrum, and the Hsi (area under the curve) increases to more than 70%. Modified Fig. 2 of reference [51]

To resolve this problem, we developed an HRV sleep index (Hsi) that detects NREM sleep based on the physiological features of cardiorespiratory regulations [51]. It is well known that during NREM sleep, breathing becomes more regular as it switches to an involuntary mode. Because respiration modulates heart rate and generates HF component at respiratory frequency, the power of the HF component concentrates on a narrower frequency band as the regularity of the respiratory cycle increases. Hsi is calculated for short-segment (~5 min) of R-R intervals (Fig. 3). First, in the power spectrum of HRV, the highest peak of HF component is detected. Second, the relative power within a frequency range of *ω* around the peak is calculated. Then, Hsi is calculated as the area under the curve (AUC) of the relative power as the function of *ω*.

**Fig. 4:**
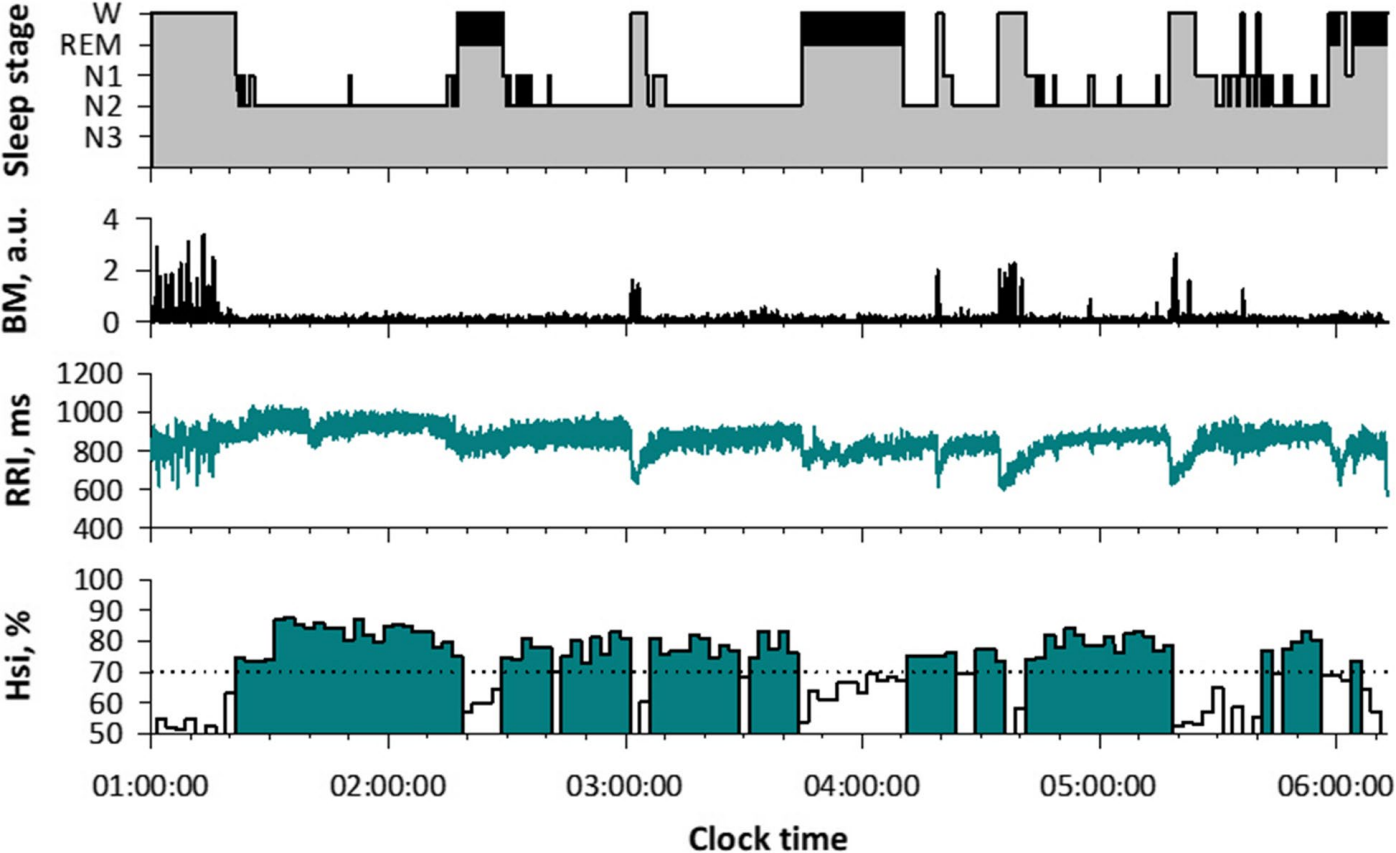
All-night hypnogram and Hsi in a healthy female subject (age, 28 years). While Hsi is below 70% during wake (W) and REM sleep (marked with black in hypnogram), it exceeds 70% in synchrony with the appearance of NREM sleep (N1, N2, and N3)

Hsi is low during wake and REM sleep and it increases during NREM sleep (Figs. 3 and 4). In a study of 141 subjects, we analyzed 11,636 consecutive 5-min ECG segments of polysomnographic data. Hsi was greater during NREM (mean [SD], 75.1 [8.3] %) than wake (61.0 [10.3] %) and REM (62.0 [8.4] %) stages. Hsi discriminated NREM sleep from wake and REM sleep with an AUC of 0.86 by receiver-operating characteristic curve analysis, which was greater than those of heart rate (0.64), peak HF power (0.75), LF/HF (0.77), scaling exponent *α* (0.77), and actigraphic body movement (0.76). With a cutoff > 70%, Hsi detected NREM segments with 77% sensitivity and 80% specificity.

Hsi has strengths not found in other HRV indices. Hsi quantifies the spectral shape of the HF component, independent of its power. This may be an advantage over other HRV indices that are dependent on age [52], respiratory rate [19], and health conditions affecting autonomic function [33]. The autonomic indices of HRV are based on an indirect or relative relationship. Although autonomic function and heart rate dynamics are known to change with sleep stage [45, 46], the change in these indices from wakefulness to sleep is continuous, not discrete, and there is no convincing physiological evidence to support the ability to distinguish light sleep from awake rest. In contrast, Hsi was developed based on solid physiological evidence of increased respiratory regularity during NREM sleep.

#### Screening of sleep apnea by HRV

Obstructive sleep apnea (SA) is a common pathophysiologic event during sleep, which affects 26% of adults, with 10% estimated to have moderate-to-severe disease [53]. Obstructive SA, however, is associated with increased risk of systemic hypertension [54, 55], atrial fibrillation and its recurrence [56–58], stroke [59, 60], sudden cardiac death during sleep [61, 62], cognitive impairment and diminished quality of life [63], and motor vehicle crashes [64]. Despite this fact, the majority of patients remain undiagnosed and miss out on treatment opportunities. Polysomnography is required for definitive diagnosis of SA, but due to its cost and limited resources, there is a need for a simple, reliable, and effective way to screen high-risk individuals who need a definitive diagnosis.

**Fig. 5:**
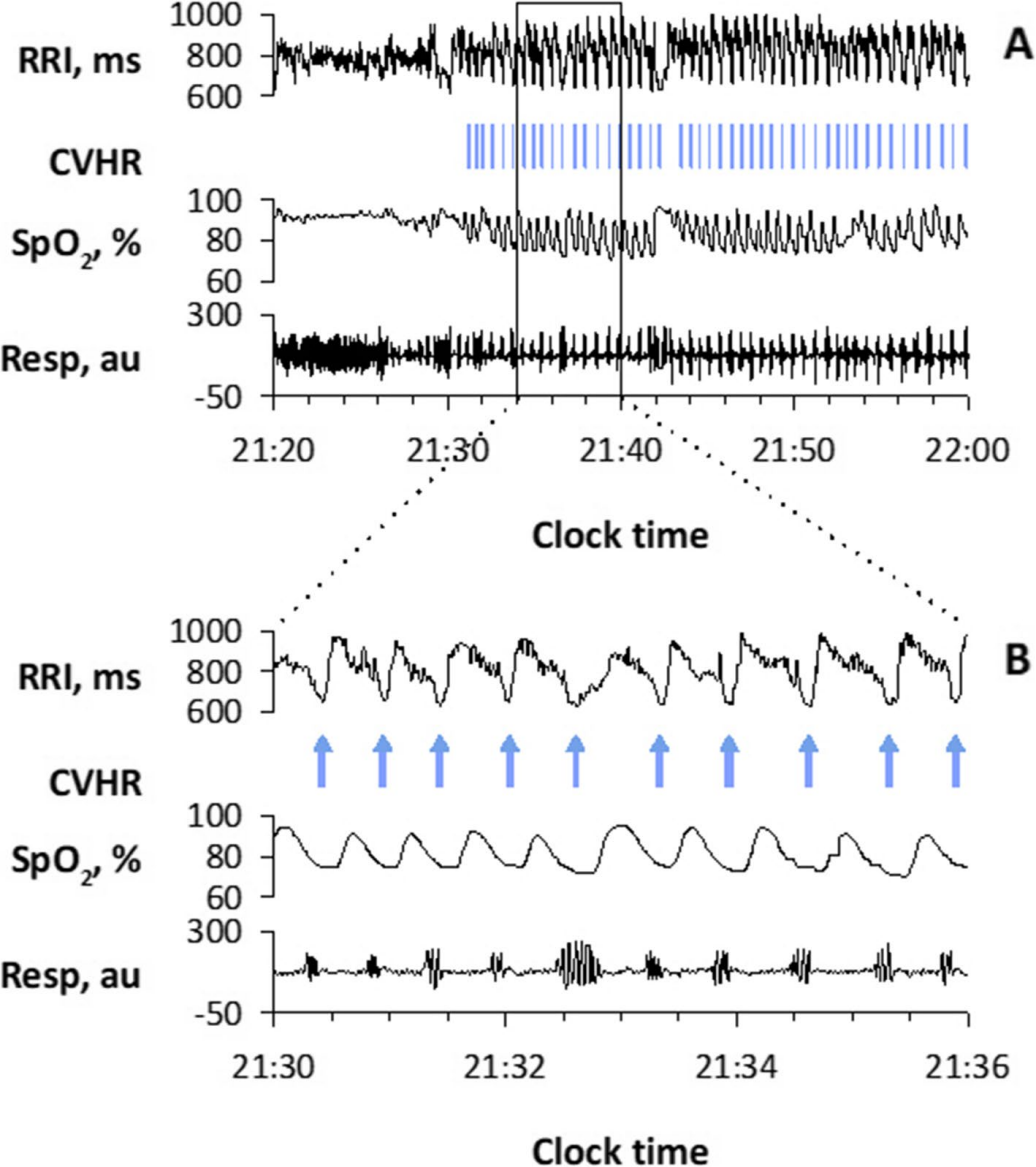
Cyclic variation of heart rate (CVHR) detected by the autocorrelated wave detection with adaptive threshold (ACAT) algorithm during a polysomnographic examination in a representative subject with obstructive sleep apnea (OSA). Panel **B** is a closer view of the data in the open box in panel **A**. Vertical bars in panel **A** and arrows in panel **B** indicate the temporal positions of detected CVHR. The ACAT algorithm detected the nadirs of cyclic dips in interbeat intervals that accompany apnea–hypopnea events. RRI, R–R interval of ECG; SpO_2_, pulse oximetric arterial blood oxygen saturation; Resp, respiration by oronasal airflow. Modified Fig. 1 of reference [68]

**Fig. 6:**
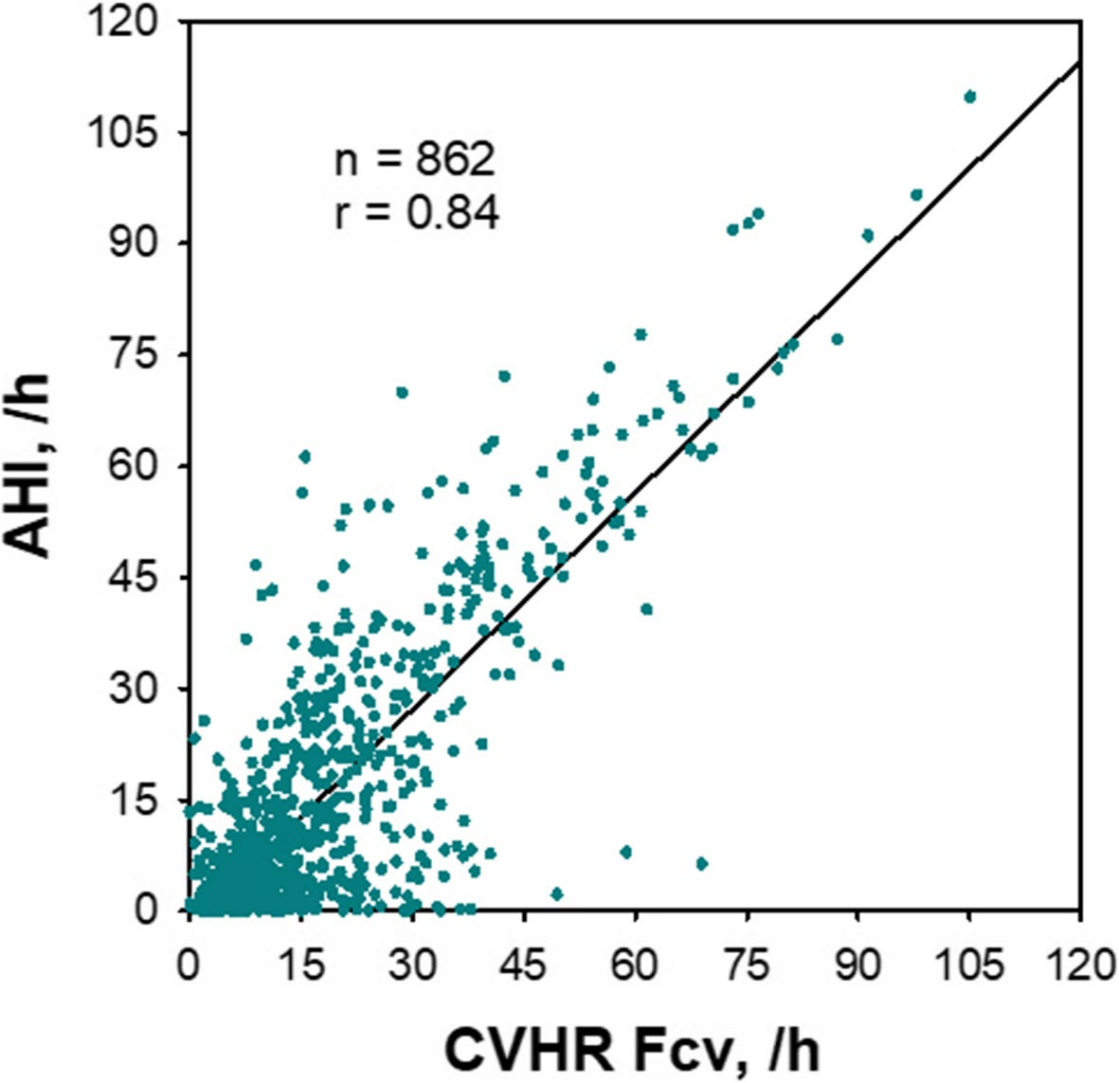
Estimation of apnea-hypopnea index (AHI) of polysomnography by the frequency of CVHR (Fcv) obtained from ECG R-R interval in 862 consecutive subjects with suspected sleep-disordered breathing. Modified Fig. 4 of reference [67]

The episodes of SA cause a characteristic pattern of HRV known as cyclic variation of heart rate (CVHR) [65]. Since CVHR appears as bradycardia during apnea and transient tachycardia during apnea cessation for individual apneic episodes, the hourly frequency of CVHR (Fcv) can be used as an estimate of the apnea-hypopnea index (AHI), which is the hourly frequency of sleep apnea and hypopnea and is an indicator of the severity of SA (Fig. 5) [66–72]. To detect CVHR in long-term HRV during sleep, we developed an automated algorism named Auto-Correlated Wave Detection with Adaptive Threshold (ACAT) [67]. In a study of 864 patients who underwent a polysomnographic study for suspected SA, the Fcv during sleep was correlated with the AHI obtained from the simultaneous polysomnography with *r* = 0.84 (Fig. 6). When Fcv > 15/h was used as the cutoff, patients with AHI > 15 were detected with a sensitivity of 83% and specificity of 88%. Detection of CVHR by ambulatory 24-h ECG (Holter monitoring) is already used clinically as a screening method for SA [73, 74]. It can also be applied to longer ECG monitoring (7 days) to reveal night-to-night variability in SA severity that is difficult to detect with other methods [75]. Furthermore, this method can be applied to pulse interval data obtained with wearable watch-shape pulse wave sensors [76], as described below.

### Evaluations of autonomic reflex functions by HRV

In order to assess autonomic reflex function, the response of peripheral organs to physiological provocation must be analyzed. In the case of baroreceptor reflex function, provocation methods such as the Valsalva maneuver, pharmacologically induced blood pressure changes, and neck suction and compression are used [77]. During long-term monitoring in daily life, the spontaneous physiological events and accidental pathophysiological events may occur, which can be used as provocations to assess autonomic reflex function. Examples of such events are ventricular premature contractions (VPCs) and SA episodes, which cause specific patterns of HRV named heart rate turbulence (HRT) and CHVR, respectively, as the autonomic responses.

#### Heart rate turbulence (HRT)

HRT refers to the phenomenon that R-R intervals show short-term fluctuations after isolated VPCs [41, 78]. In normal subjects, HRT consists of an initial brief shortening (turbulence onset, TO) followed by a gradual elongation (turbulence slope, TS) before it returns the pre-VPC baseline [79]. The physiological mechanism of the initial shortening is a transient vagal inhibition in response to the missed baroreflex afferent input caused by hemodynamically inefficient ventricular contraction and that of the subsequent gradual elongation is a reflex vagal activation caused by a sympathetically mediated overshoot of arterial pressure. Therefore, the HRT pattern is blunted in patients with reduced baroreflex, and HRT measurement provides an indirect assessment of baroreflex.

HRT is measured by averaging R-R interval segments (from 2 intervals before to 15 intervals after) of >5 isolated VPCs. The VPCs adequate to HRT measurement are limited to noninterpolated VPCs with prematurity of > 20% and compensatory pause of > 120%, bounded by 17 preceding and 15 subsequent continuous sinus rhythm cycles. TO is quantified as the percentage of R-R interval decrement, i.e., the average of two R-R intervals immediately following the compensatory pause minus the average of two R-R intervals immediately preceding the VPC coupling interval. TS is measured as the maximum positive regression slope assessed over any 5 consecutive sinus rhythm R-R intervals within the first 15 sinus rhythm R-R intervals after the VPC. Both TO and TS are used as indices of vagal baroreflex function. In clinical studies, TO < 0% and TS > 2.5 ms/R-R interval are considered normal.

Several prospective studies confirmed that abnormal HRT is a powerful predictor of post-MI mortality [41, 78], but this method is applicable only to ECG recordings under sinus rhythm including > 5 adequate isolated VPCs. In the Allostatic State Mapping by Ambulatory ECG Repository (ALLSTAR) [80–82], big data of HRV in patients undergone Holter 24-h ECG monitoring in Japan, isolated VPCs meeting with the requirements for HRT were found only in 158,933 (39.8%) out of 399,458 recordings in adult patients aged > 20 years.

#### Amplitude of CVHR (Acv)

**Fig. 7:**
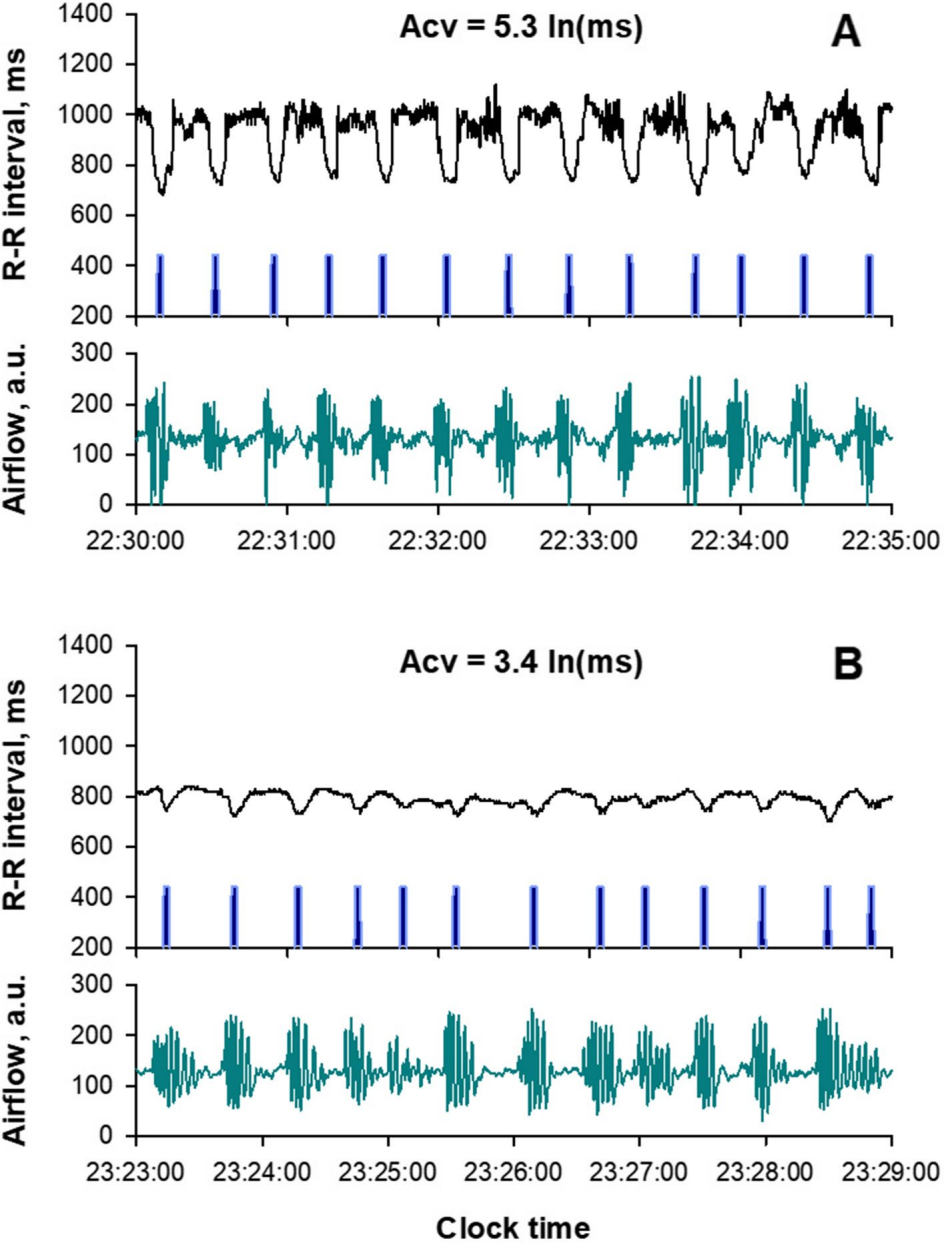
CVHR associated with sleep apnea episodes in a representative patient with normal vagal reflex function (**A**) and a patient with moderately impaired vagal reflex function (**B**). The blue bars indicate the temporal location of the dip in the R-R interval caused by CVHR. Airflow detected by mouth-nose thermistor shows intermittent apnea or hypopnea in both patients, but the response of the R-R interval, or CVHR, is blunted in patient B compared to patient A. This is reflected in the difference in the amplitude of the CVHR (Acv), 3.4 vs. 5.3 ln (ms)

As mentioned earlier, the frequency of CVHR (Fcv) reflects the frequency of SA episodes [67], but CVHR itself is a heart rate response provoked by spontaneous apneic/hypoxic load caused by SA. CVHR consists of bradycardia during apnea and abrupt, transient tachycardia during the cessation of apnea [66]. The mechanism of the bradycardia during apnea is thought to include an increase in cardiac vagal activity resulting from the combined effect of cessation of breathing and hypoxemia [83], and that of the tachycardia during apnea cessation is thought to include sympathetic activation by hypoxia and cardiac vagal suppression associated with arousal, baroreceptor unloading, and respiratory recovery. In a study of 400 patients with SA, Guilleminault et al. [66] observed the absence of CVHR in a subgroup of SA patients with impaired cardiac autonomic function (heart transplants, autonomic neuropathy, and Shy-Drager syndrome). They also observed that in SA patients with normal cardiac autonomic function, intravenous atropine blocked CVHR by eliminating the bradycardia component. These indicate that the blunted CVHR can be used as an index of impaired cardiac vagal reflex function (Fig. 7).

**Fig. 8:**
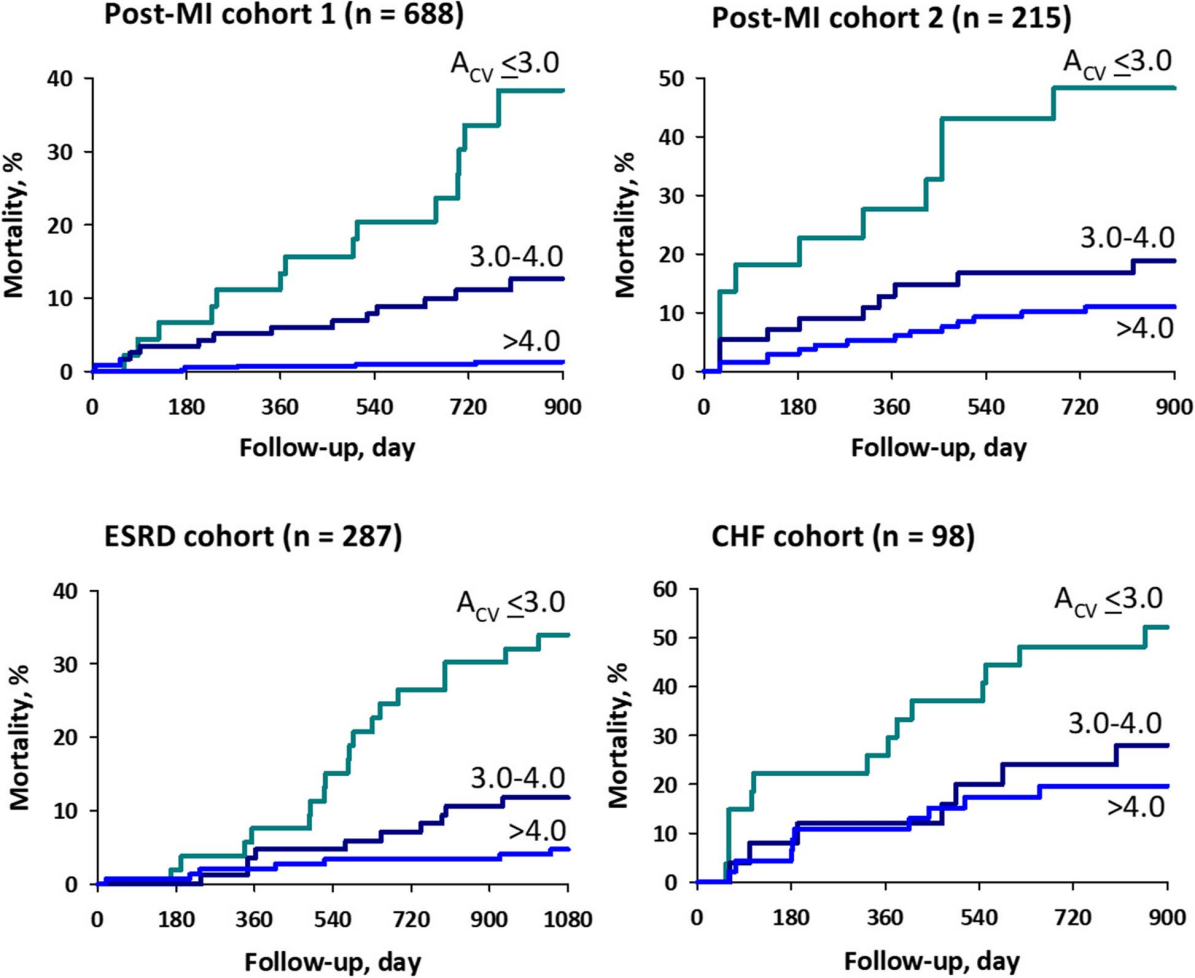
Mortality probabilities in four cohorts of patients stratified by the same cut-off values of the amplitude of CVHR (Acv; 4.0 and 3.0). CHF, congestive heart failure; ESRD, end-stage renal failure; MI, myocardial infarction. Modified Fig. 3 in reference [73]

We studied the predictive value of blunted CVHR observed in ambulatory ECG in 717 post-MI patients (mortality, 6% during median follow-up for 25 months), 220 post-MI patients (25.5% mortality during 45 months), 299 patients with end-stage renal disease (ESRD) on chronic hemodialysis (28.1% mortality during 85 months), and 100 patients with chronic heart failure (CHF; 35% mortality during 38 months) [73]. CVHR was detected by the ACAT algorithm from night-time ECG and Fcv was measured as hourly frequency of CVHR during estimated sleep period. The magnitude of CVHR was measured as the mean log amplitude (Acv) of CVHR only if at least four CVHRs were observed per night, which was met by > 96% of patients in all cohorts. Although Fcv did not predict mortality in any cohort, decreased Acv was a powerful predictor of mortality in all cohort (Fig. 8). The prognostic value of Acv was independent of age, gender, diabetes, beta-blocker therapy, left ventricular ejection fraction, sleep-time mean R-R interval, and Fcv. Along with earlier studies [66, 83], this indicates that Acv obtained from nocturnal ECG is an indicator of cardiac vagal reflex function, and its decline is an independent predictor of increased mortality risk, common in post-MI, ESRD, and CHF patients.

Although the measurement of Acv requires the presence of CVHR, it can be obtained even in individuals without clinically significant frequency of CVHR (Fcv ≥ 5/h). In the above study [73], the Acv calculated when ≥ 4 CVHRs were observed per night had predictive power. The value of Acv, however, shows a large variation when Fcv is very low, thereby reducing the prognostic ability. This problem can be improved by adjusting the cutoff value of Acv according to the value of Fcv [82]. The high applicability ratio of Acv is the advantage of Acv compared to HRT, which can only be applied to Holter ECG recordings (< 40%) that contain adequate VPCs.

### Is pulse rate variability a surrogate of HRV?

In recent years, the widespread use of wearable watches equipped with photo-plethysmograph (PPG) sensors has facilitated the measurement of pulse wave signals in daily life, and as a result, many studies have been published that attempt to use pulse rate variability (PRV) as a substitute for HRV. However, there are serious pitfalls in applying the classical LF-HF framework to PRVs in daily activities. It is not only a problem of applying the classical framework obtained under controlled conditions to data under free-running condition, but also a problem caused by the essential difference between PRV and HRV.

**Fig. 9:**
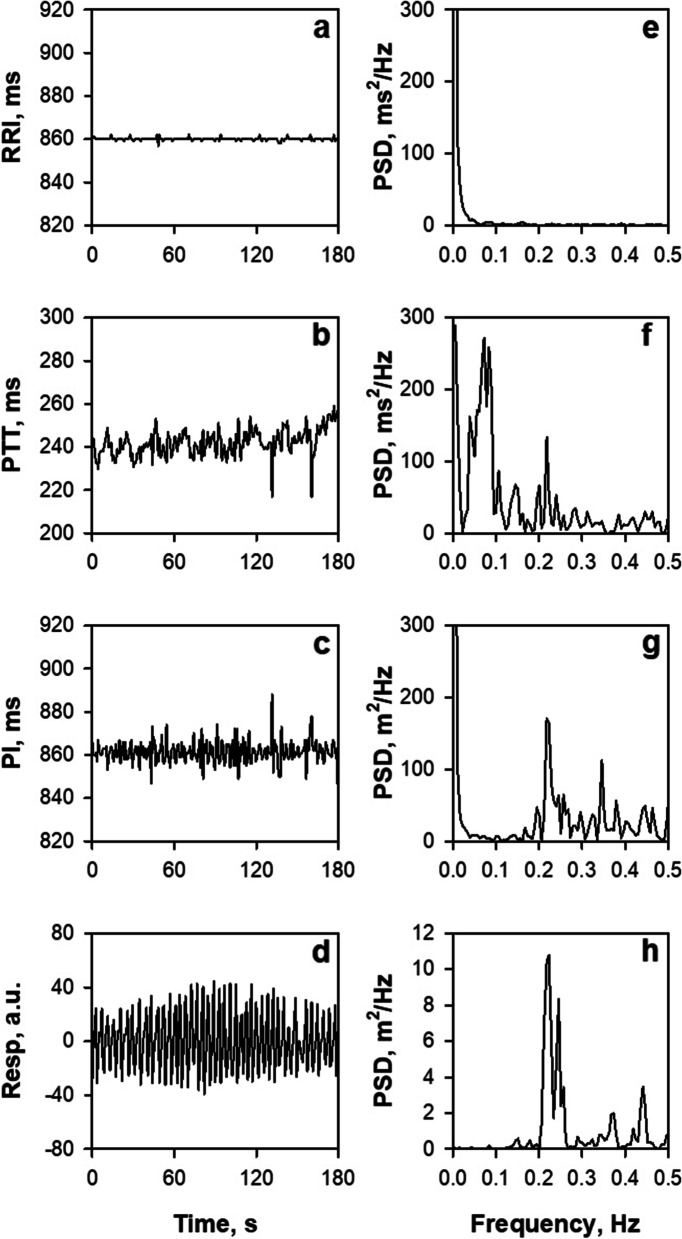
Trend grams (**a**‑**d**) and power spectra (**e**‑**h**) of R-R interval (RRI), pulse transit time (PTT), pulse interval (PI), and respiration (Resp) obtained by simultaneous recordings of ECG, finger-tip photoplethysmography (PPG), and nose-tip thermistor respiration in a patient with an implanted cardiac pacemaker with a fixed pacing rate (70 bpm). PTT was measured as time from ECG R wave to PPG presystolic foot point of each beat and PI as the interval between the foot points of consecutive pulse waves. PSD, power spectral density. Modified Fig. 1 in reference [84]

PRV should be recognized as a different biomarker than HRV. HRV is one of the sources of PRV, but HRV is not the only source of PRV. The process from ECG R wave to PPG pulse wave involves several transformation steps of physical properties, such as those of electromechanical coupling and conversions from force to volume, volume to pressure, pressure impulse to wave, pressure wave to volume, and volume to light intensity, and many sources of fluctuation can modulate each of these processes [84]. In fact, there is evidence that shows discrepancy between PRV and HRV, such as that demonstrating the presence of PRV in the absence of HRV [85], differences in PRV with measurement sites [86, 87], and differing effects of body posture and exercise between them [88]. Figure 9 shows our recent observation in an adult patient with an implanted cardiac pacemaker, indicating that fluctuations in R-R intervals, pulse transit time, and pulse intervals are modulated differently by autonomic functions, respiration, and other factors [84].

The HF of PRV is not the same as the HF of HRV, nor is LF or LF/HF. PRV may contain useful biomedical information, but finding it requires an approach beyond the classical framework. For example, nocturnal PRV can be used to screen for SA by detecting cyclic variation of pulse rate (CVPR) [76]. In 41 patients who underwent diagnostic polysomnography (PSG) for SA, PPG was recorded simultaneously with a wearable watch device. The median (IQR) AHI of patients was 17.2 (4.4‑28.4), and 22 (54%) patients had moderate-to-severe SA (AHI ≥ 15). SA episodes were accompanied by CVPR, a characteristic pattern of PRV similar to the CVHR of HRV. The hourly frequency of CVPR detected by the ACAT algorithm correlated with AHI (*r* = 0.81), but none of the time-domain, frequency-domain, or non-linear indices of PRV showed a significant correlation. The CVPR frequency (> 11/h) was able to discriminate patients with moderate-to-severe SA (AHI > 15) with 82% sensitivity, 89% specificity, and 85% accuracy. The classification performance was comparable to that obtained when the ACAT algorithm was applied to HRV during the PSG.

## Conclusions

HRV analysis using the LF-HF framework has contributed significantly to the widespread use of HRV to assess autonomic function, but this classical framework should only be used for HRV under tightly controlled conditions.

This framework is inappropriate for the interpretation of HRV and PRV measured under free-running conditions, such as those obtained with wearable sensors, and does not adequately capture the useful information contained therein. In order to use HRV and PRV for the assessment of autonomic function in daily life and to extract other useful biomedical information from them, it is necessary to research and develop new means of capturing them that go beyond the classical framework. In this review, several studies were presented as examples suggesting such approach.

## Data Availability

Not applicable
